# Anger Rumination and Mindfulness: Mediating Effects on Forgiveness

**DOI:** 10.3390/ijerph18052668

**Published:** 2021-03-06

**Authors:** Raquel de la Fuente-Anuncibay, Ángela González-Barbadillo, Delfín Ortega-Sánchez, Nuria Ordóñez-Camblor, Juan Pablo Pizarro-Ruiz

**Affiliations:** 1 Department of Education Sciences, Faculty of Education, University of Burgos, 09001 Burgos, Spain; raquelfa@ubu.es (R.d.l.F.-A.); agb0067@alu.ubu.es (Á.G.-B.); 2 Department of Specific Didactics, Faculty of Education, University of Burgos, 09001 Burgos, Spain; dosanchez@ubu.es; 3 Department of Health Sciences, Faculty of Health Sciences, University of Burgos, 09001 Burgos, Spain; nordonez@ubu.es

**Keywords:** anger rumination, mindfulness, forgiveness, mediation

## Abstract

(1) Background: Different investigations relate mindfulness practice as a strategy to cope with and improve negative repetitive thinking states and forgiveness. (2) Methods: The aim is to analyze the mediating processes of mindfulness as a trait and the changes in the anger rumination on forgiveness. This sample comprised 264 undergraduate students (*M* = 24.13 years, *SD* = 11.39). The instruments used were the Anger Rumination Scale (ARS), the Five Facet Mindfulness Questionnaire (FFMQ) and the Heartland Forgiveness Scale (HFS). For data analysis, the spillover effect was calculated using 10,000 bootstrap samples for the bootstrap confidence intervals (CI). (3) Conclusions: The results confirm that the relationship between mindfulness practice and forgiveness is mediated by changes in mindfulness trait and anger rumination. Given the results obtained, it is considered appropriate to extend the study to samples from other countries, as well as to contexts of depressive rumination or anxiety.

## 1. Introduction

Rumination is a psychological construct that refers to recurrent, conscious thoughts about a particular subject [1]. There is no single definition, but there is a consensus that it is a non-adaptive coping strategy in which uncontrollable and repetitive thoughts with a negative connotation appear and focus on the negative mood, its causes, meanings and consequences [2,3].

This term was originally associated with sadness and depression, as people with a tendency to ruminate on its symptoms, causes and consequences suffered longer and more intensely from the effects of the depressed state [2,4]. Today, accumulated evidence shows that rumination and depressive symptoms influence each other in a bidirectional and recursive way [5]. Rumination of interpersonal aggression can reactivate the experience as if it were being lived again, causing the person to experience again the emotions associated with the event [6]. It has also been associated with aggressive behavior [7,8]. Although ruminant individuals may perceive this pattern of thinking as a productive problem-solving strategy, it contributes to a downward spiral of negative affect and further deregulated behavior [9,10]. Nolen-Hoeksema and Watkins [11] note that it is a common factor underlying the development of multiple forms of psychopathology. In this respect, rumination has been associated with anxiety problems, insomnia, eating behavior, cardiovascular disease or social phobia in adult and adolescent populations [7,12,13,14,15].

Roberts, Moberly, Cull, Gow, Honeysett and Dunn [16] conclude that specificity of thought content may be an important determinant of whether goal-focused rumination has helpful or unhelpful effects.

### 1.1. Anger Rumination

Research has defined different types of rumination as depressive, anger, etc. based on the predominant negative content of repetitive thoughts [3,15]. Pérez, Redondo and León [17] describe anger as a universal primary emotion that involves physiological activation and a characteristic facial expression accompanied by feelings of anger and that appears when a goal or need is not achieved or harm is received. Anger only appears to be maladaptive when it occurs in an inappropriate environment or is produced in excess [18]. Some cross-cultural studies [19] indicate the stability of physiological symptoms in anger such as dysplastic arousal, ‘feeling hot’, muscle tension or increased blood pressure due to activation of the sympathetic system.

Anger rumination has been described as the tendency to repeatedly think about past experiences that caused negative affect in the form of this emotion. It is partly responsible for the maintenance and intensification of anger, thus exacerbating the negative consequences of anger [3]. Rumination follows anger as an attempt to ‘solve’ the problem. Thus, people in a state of anger seek causes or solutions to that emotion, which leads to rumination. When the problem seems uncontrollable or unsolvable, in particular, they may have difficulty disconnecting from rumination. Therefore, it can be conceptualized as a broad formulation of the problem, in which people seek information that will help them generate and evaluate explanations for an angry experience [20].

The present paper focuses on this type of rumination, conceived as a non-adaptive strategy of anger regulation [20] and defined as the tendency to focus on the past and then continue to have recurring unintentional thoughts about episodes of anger, about perceived injustices and fantasize about revenge [3,21]. While depressive rumination has been associated with a worsening of depressed mood, anger rumination is negatively associated with well-being and specifically intensifies anger, aggressive and suicidal tendencies, predicts physical and verbal aggression, hostility, displaced aggression and facilitates the formation of negatively distorted interpretations of ambiguous events [7,22,23,24,25,26,27].

### 1.2. Forgiveness

Research on forgiveness in psychology had a boom in the 1990s, driven in part by positive psychology, as it is considered a human strength that contributes to the overall well-being of the individual [28]. Despite the different definitions, there is a strong consensus that forgiveness is an adaptive behavior linked to psychological well-being and that it includes the renunciation of anger and resentment [29,30,31,32]. In fact, forgiveness itself can promote healthy behaviors [33].

Unforgiveness has been positively correlated with indicators of stress and psychopathology [29,34,35]. Forgiveness is defined within the framework of a perceived transgression, such that one’s responses to the transgressor, the transgression, and the aftermath of the transgression are transformed from negative to neutral or positive [36]. The source of a transgression, and thus the object of forgiveness, may be oneself, another person or people, or a situation that is considered beyond anyone’s control (e.g., illness, “fate” or natural disaster).

When people forgive, they recognize that a transgression has occurred and, more specifically, it is their thoughts, feelings, motivations and behaviors that change to reframe the transgression, so that their responses to it are no longer negative.

### 1.3. Relationship between Anger Rumination and Forgiveness

Recent research suggests that reducing rumination may facilitate the cognitive decision to forgive [37], that rumination negatively predicts forgiveness, and that the possibility of forgiveness can be increased by reducing rumination [38,39,40]. These results have also been found in cross-cultural research [41]. Some authors [37,42,43] note that, among university students, rumination is considered one of the most important factors in interpersonal and intrapersonal forgiveness. 

Not forgiving proves to be one of the most important sources of stress in an individual’s life, and in turn, forgiveness is recognized as an effective coping mechanism that can be used to deal with this stressful state of mind [43]. Numerous studies conclude that forgiveness of others and forgiveness of oneself are predictors of people’s physical and psychological health: they reduce depression, stress and feelings of shame and improve life satisfaction and levels of subjective happiness [36,44,45,46,47]. When people reflect on hurtful events, they re-experience and immerse themselves in negative thoughts and emotions, making them less likely to forgive [40]. Freedom from reflective thoughts can help an individual to assess transgression in a wider perspective and, therefore, decide to forgive rather than focus only on their pain, worry and negative mood [48].

In the present study, we follow the conception of forgiveness by Thopmson et al. [36] and Berry et al. [49], according to which forgiveness is understood as a personality trait that alludes to the tendency to forgive across time and situations, that is, dispositional forgiveness. 

Measures of dispositional forgiveness are especially useful for studying the relationships of forgiveness with other psychological constructs [49], as the literature on the topic indicates that dispositional forgiveness scores tend to be related to mental health and well-being, whereas specific transgressions forgiveness assessments tend not to be significantly related to either mental health or well-being [36].

While the previous literature is clear that rumination is strongly and negatively associated with forgiveness, the mechanisms of action of this relationship are less studied in the context of anger rumination.

### 1.4. Relationship between Mindfulness, Anger Rumination and Forgiveness 

“Paying attention in a particular way: on purpose, in the present moment and non-judgmentally” is one of the most popular definitions of mindfulness, a practice rooted in Buddhist philosophy. With regard to the field of intervention, work in recent years has reported benefits from mindfulness practice, with clinical and health studies being the most extensive, mainly in the treatment of anxiety, depression and stress reduction [50,51,52], although, because of the benefits, its practice is increasingly being extended to business, sports or educational settings [53,54,55,56,57]. Most mindfulness-related research with university students has focused on therapeutic settings, focusing on student health and well-being, depression reduction, anxiety, improved academic performance, and empathic processes [58,59,60]. 

Forgiveness is positively related to the development of empathy and perspective taking [61,62]; both constructs would benefit from the cognitive flexibility provided by mindfulness training [63]. Components of mindfulness such as non-reactivity, non-judgment and perspective taking can help with forgiveness. One aspect of mindfulness that was positively related to forgiveness of the other, specifically in the area of partner infidelity, was non-reactivity [64]. This component of mindfulness seems to be especially important to allow observation of experiences from a psychological distance that allows greater access to information [65], while at the same time it would imply not adhering to negative emotions, such as anger, and would also facilitate the transformation of the negative response to transgressions into a neutral or positive response.

Similarly, mindfulness could facilitate the decision to forgive through perspective-taking and recognition that one is in a state of unforgiveness, along with an understanding that such a state is causing harm or discomfort to oneself [66].

Some studies have shown that mindfulness has an effect on rumination, mediating the effects of mindfulness-based interventions, and Ramel et al. [67] found that reduction in rumination after mindfulness training explained the reduction in anxious and depressive symptoms. However, research analyzing the effects of mindfulness practice on anger rumination is more scarce. Borders et al. [68] used a general, non-rage-specific rumination questionnaire to study the effect of mindfulness on anger, hostility and verbal and physical aggression through rumination, and their results concluded that rumination partially mediated the effect of mindfulness on anger and aggression. Along these lines, recent work [69] shows that mindfulness training reduces anger, hostility and aggression both physically and verbally, through reductions in anger rumination, although the mechanisms of these effects are not clear. Contreras et al. [70] studied the attenuation of anger rumination by the willingness to forgive, but it may be that anger rumination induced people to forgive less. McCullough et al. [71] analyzed journal entries that showed the relationship between variables to be in both directions, with anger rumination having a stronger effect on reducing forgiveness than forgiveness on reducing anger rumination. Mindfulness-based interventions are specifically useful in disorders characterized by anger rumination [48]. More specifically, some studies indicate that the mindfulness trait is less able to predicts the rumination and anger traits [68,69,72]. However, the underlying processes that explain how mindfulness and anger rumination are linked to forgiveness remain unknown. Few studies have focused on whether forgiveness is related to anger and aggression, and no studies found studies that explore the mechanisms between these three constructs. 

The objectives of this work focused on analyzing the mediating mechanisms by which the mindfulness practice in leisure and non-therapeutic contexts reduces anger rumination and increases dispositional forgiveness towards oneself, towards others and towards negative situations beyond people’s control, in a sample of university students. Three objectives were set: the first one was to evaluate the spillover effect of mindfulness practice on dispositional forgiveness from two mediators: changes in mindfulness traits and anger rumination. The second objective was to study the spillover effects of each of the factors of anger rumination in order to determine which ones predict dispositional forgiveness through the mindfulness practice. Once the significant anger rumination factors are known, the third objective aimed to analyze its spillover effects and the mindfulness trait on each of the forgiveness factors (towards oneself, towards others and towards situations out of control) through the mindfulness practice.

Different research has shown the relationship between anger rumination and the willingness to forgive, and it has also been shown that mindfulness practice has effects on the reduction of rumination, so our objective was focused on understanding the mediating mechanisms between these three constructs.

## 2. Materials and Methods

### 2.1. Participants 

A cross-sectional study was designed and applied to student volunteers from the Faculty of Psychology at Sheffield Hallam University, Sheffield-South Yorkshire, England. The sample consisted of 264 students, with a gender distribution of 48 males and 216 females with a mean age of 24.13 years (standard deviation (*SD*) = 11.39). The bias in the gender distribution was due to the representation of this type of study among the university population in European countries. According to Organisation for Economic Cooperation and Development (OECD) reports [73], this bias is 68% in Ireland in the area of education, rising to 79% and 75% respectively, in countries such as the United Kingdom and Ireland in the areas of health and social services. The choice was made to study a representative sample of the population rather than a more homogeneous sample. 

The criteria for dividing the sample on the mindfulness practice were set in two groups: those who had never practiced and those who practice in informal contexts, that is, outside closed programs with a predetermined number of sessions in non-therapeutic contexts.

### 2.2. Procedure and Instruments

The informed consent of the participants was obtained, as well as approval by the Bioethics Committee of the University of Burgos (IR 15/2018). Confidential treatment of the data collected was guaranteed. The questionnaires were completed in about 25–30 min, and the information was collected in a single phase.

The instruments used were the Five-Facet Mindfulness Questionnaire (FFMQ), designed to measure mindfulness trait or tendency to be more aware in daily life [74]. The scale was constructed from a factor analysis of five scales: Kentucky Inventory of Mindfulness Skills (KIMS), Freiburg Mindfulness Inventory (FMI), Mindfulness Questionnaire (MQ), Mindful Attention Awareness Scale (MAAS), and the Cognitive and Affective Mindfulness Scale (CAMS). It consisted of 39 items with five subscales: ‘observing’ (ability to perceive and recognize internal or external stimuli; ‘describing’ (labelling the living experience with words); ‘acting consciously; not judging the internal experience’ (equable view of the perceived thoughts, sensations or emotions); and ‘not reacting to the internal experience’ (refers to the distance from the experience and a time span in which one does not react to the stimulus). The response scale consisted of five alternatives from 1 (“never or very rarely true”) to 5 (“very often or always true”). It had adequate reliability, and convergent and discriminatory validity [74]. 

The Anger Rumination Scale (ARS) by Sukhodolsky et al. [3] was used to obtain data on anger. This scale measures the tendency to focus attention on states of anger, recall past experiences of anger, and think about the causes and consequences of anger episodes. It consists of 19 items with 4 subscales: angry or rage memories, understanding the causes of the anger, thoughts after the anger and thoughts of revenge. The response scale consists of four alternatives from 1 (“rarely”) to 4 (“almost always”). The higher the score, the greater the tendency to ruminate on anger. In its initial validation, it displayed an adequate internal consistency with Cronbach’s alpha ranging from 0.72 to 0.86 and a good test-retest reliability of one month (rxy = 0.77). 

The Heartland Forgiveness Scale (HFS) developed by Thompson et al. [36] was used to assess forgiveness. It consists of 18 self-reporting items that measure a person’s willingness to forgive (i.e., general tendency to forgive), rather than the forgiveness of a particular event or person. It consists of three subscales: self-forgiveness, forgiveness towards others and situation-forgiveness, which shows how one tends to forgive negative circumstances, events or situations that are beyond one’s control such as illness or natural disaster [36]. The response scale consists of seven alternatives from 1 (“almost always false of me”) to 7 (“almost always true of me”). It had an appropriate consistency with a Cronbach’s alpha ranging from 0.72 to 0.87. There is evidence to support the validity of the HFS subscale construct with samples from university students and non-students [36,75]. 

Table 1 shows the reliability (Cronbach’s alpha), mean, standard deviation, skewness and kurtosis of all the dimensions used. In the Appendix A (Table A1, Table A2 and Table A3), the descriptive data (mean, standard deviation, minimum and maximum scores, and percentage of the sample answering each response option) corresponding to all the items of the questionnaires can be consulted.

### 2.3. Data Analysis

In order to respond to the first objective, the PROCESS macro for SPSS developed by Hayes [76] was used, specifically Model 6, which postulates a mediation model with two mediating variables. This procedure was used as an analytical strategy to assess the spillover effect of mindfulness practice on dispositional forgiveness through the mediating process of two mediators: mindfulness trait and anger rumination. The spillover effect was calculated using 10,000 bootstrap samples for the bias-corrected bootstrap confidence intervals (CIs). A spillover effect is considered statistically significant if the established CI (95% CI) does not include the value 0. If the 0 value is included in the CI, the null hypothesis is that the bootstrap effect is equal to 0, that is, there is no association between the variables involved [76]. Previously, the Durbin–Watson (*DW*) statistic was used to test the independence between the residuals of the variables, and independence could be assumed (values between 2.05 and 1.86).

For the second and third objectives, the PROCESS macro for SPSS developed by Hayes [76] was also used. As in the first objective, the spillover effect was calculated using 10,000 bootstrap samples for the bootstrap confidence intervals.

## 3. Results

Firstly, the influence of the mindfulness practice on dispositional forgiveness was analyzed. The results of the total effect of mindfulness practice (IV) on dispositional forgiveness (DV) confirm that mindfulness practice increases levels of forgiveness towards oneself, towards others and towards negative situations which are beyond the person’s control (H1: *B* = 0.966, *p* < 0.0001).

**Figure 1:**
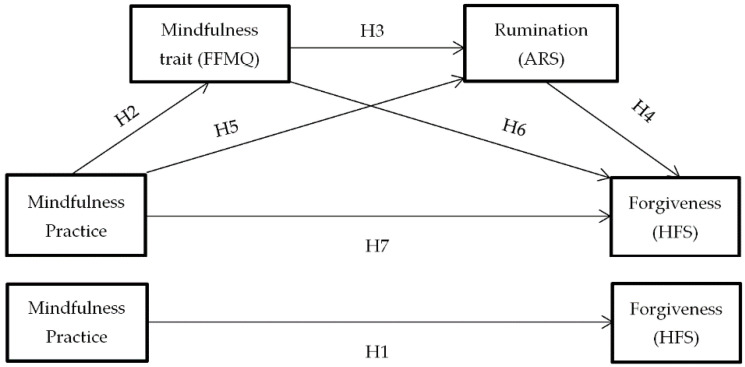
Hypothesized mediation model: Spillover effect of mindfulness practice on forgiveness. H1–H7: Hypothesis 1–Hypothesis 7.

However, in this research it is argued that this relationship is not direct, but is mediated by the changes that mindfulness practice (X) predicts in dispositional mindfulness (M1 = mediator 1) and these, in turn, will predict a decrease in anger rumination (M2 = mediator 2), thus causing an increase in dispositional forgiveness (Y) Figure 1. In the presence of these two mediators, the influence of mindfulness practice on forgiveness would no longer be significant.

**Figure 2:**
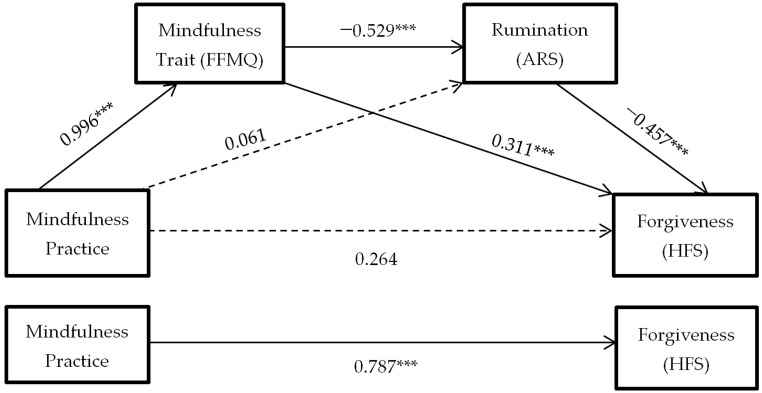
Hypothesized mediation model: spillover effect of mindfulness practice on forgiveness through mindfulness trait and rumination. Total effect of independent variable on dependent variable. *** = *p* < 0.001.

As can be seen in Figure 2, the hypothesized mediation model is confirmed: the mindfulness practice predicts dispositional mindfulness (H2: *B* = 0.996, *p* < 0.0001), it predicts decreased levels of anger rumination (H3: *B* = −0.529, *p* < 0.0001), and it predicts increased dispositional forgiveness (H4: *B* = −0.457, *p* < 0.0001). Furthermore, the influence of mindfulness practice on forgiveness ceases to be significant in the presence of these two mediators (H7: *B* = 0.264, *p* > 0.05).

**Figure 3:**
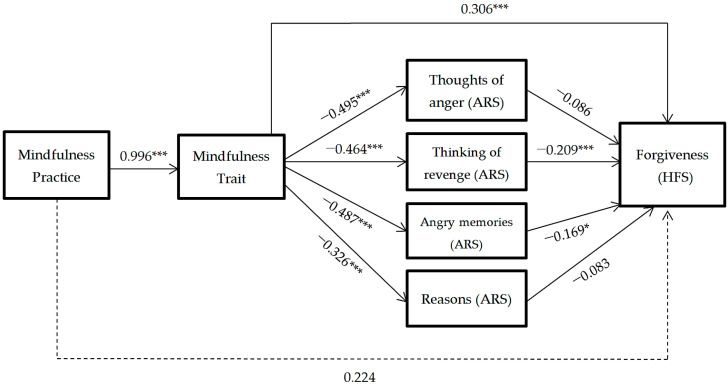
Hypothesized mediation model: spillover effect of mindfulness practice on forgiveness through mindfulness trait and rumination factors. * = *p* < 0.05, *** = *p* < 0.001.

To analyze the mechanisms through which anger rumination influences forgiveness, a mediation model was developed with each of the anger rumination dimensions as simultaneous (non-sequential) mediators: thoughts of being angry, thoughts of revenge, angry memories, and understanding the causes Figure 3.

The results reflect that only the thoughts of revenge and angry memories factors (ARS) are significant in predicting forgiveness. Thus the mindfulness practice predicts dispositional mindfulness, and it predicts the decrease of thoughts of revenge and angry memories, which predicts the increase of levels of forgiveness towards oneself, towards others and towards negative situations that are beyond a person’s control. In this mediation model, the direct effect of mindfulness practice on forgiveness disappears (*p* > 0.05).

**Figure 4:**
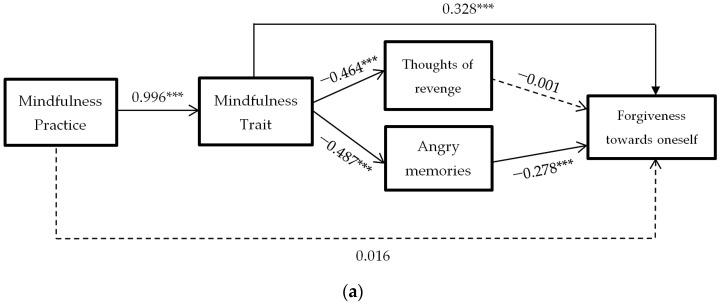

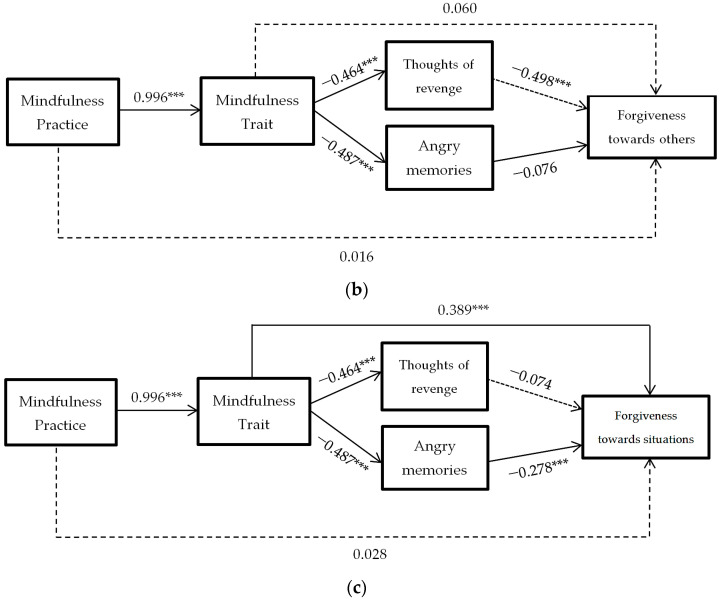
Hypothesized mediation model: spillover effect of mindfulness practice on forgiveness to oneself (**a**), to others (**b**) or to situations (**c**) through mindfulness trait, and thoughts of revenge and angry memories. *** = *p* < 0.001

In response to the third objective, three models of mediation are carried out, similar to the previous ones, but in which DV (dependent variable) is the dimensions of forgiveness towards oneself, towards others, and towards negative situations outside the person’s control Figure 4. 

As can be seen, the results indicate that thoughts of revenge mediation is significant in forgiving oneself and in negative situations out of control. Furthermore, in these two mediation models, the mindfulness trait predicts increased forgiveness without the need for decreased rumination.

On the other hand, the mediation of angry memories is only significant in forgiveness towards others. This time, the mindfulness trait does not directly predict forgiveness towards others, the decrease of angry memories being necessary for the spillover effect on forgiveness to be significant.

In all three models the mindfulness practice stops predicting increased forgiveness when the mediating variables are considered.

## 4. Discussion

Studies analyzing the mediating effects of mindfulness on rumination and forgiveness are scarce, however, some research points to underlying explanations for this phenomenon.

The study of the influence of cognitive variables such as anger and mindfulness rumination and emotional affectation is particularly interesting from an educational and psychological point of view, since, unlike other psychological variables such as personality traits, they are modifiable through cognitive-behavioral intervention techniques. 

Recent research suggests that reducing rumination may facilitate forgiveness [37]. Forgiveness explained a significant amount of variation in ARS (anger rumination) scores, even when the thoughts of revenge subscale was removed. This subscale is relevant to aggressive behavior, but its relationship to forgiveness is not direct [70].

This research has started from the conception of rumination by Sukhodolsky et al. [3], who define it as the tendency to experience involuntary and repetitive thoughts following an episode of anger or rage. These researchers hypothesize that anger rumination may produce behavior in the realm of aggression. 

As can be seen in Figure 2, it can be concluded that the mindfulness practice itself does not directly predict changes in anger rumination (H5: *B* = 0.264, *p* > 0.05), but that changes in the mindfulness trait are necessary for anger rumination to decrease. However, changes in the mindfulness trait do predict levels of forgiveness (H6: *B* = 0.311, *p* < 0.0001) without the need for a decrease in anger rumination. 

The dimensions of anger rumination that best predict increased forgiveness through the mindfulness practice are thoughts of revenge and angry memories. 

Regarding the third objective, the results conclude that the mindfulness practice predicts modifications in mindfulness traits (*B* = 0.996, *p* < 0.0001), and these predict a decline in thoughts of revenge (*B* = −0.464, *p* < 0.0001) and in angry memories (*B* = −0.278, *p* < 0.0001). This decline in angry memories predicts an increase in forgiveness towards oneself (*B* = −0.278, *p* < 0.0001) and towards situations beyond one’s control (*B* = −0.278, *p* < 0.0001). The decrease in thoughts of revenge predicts an increase in forgiveness towards others (*B* = −0.498, *p* < 0.0001). The mediation is total in all, with the direct effect of mindfulness practice on forgiveness factors disappearing (*B* = 0.016, *B* = 0.341 and *B* = 0.028, *p* > 0.05) when considering the mediation of mindfulness traits and anger rumination (thoughts of revenge and angry memories). 

In addition, the mindfulness trait directly predicts forgiveness to oneself (*B* = 0.328, *p* < 0.0001) and to situations beyond a person’s control (*B* = 0.389, *p* < 0.0001). However, it does not do so in forgiveness to others (*B* = 0.060, *p* > 0.05), with decreased rumination being necessary to increase forgiveness to others. As these results seem to indicate, decreasing rumination would help to increase forgiveness towards oneself and towards situations out of control. Therefore, it seems to enable the conclusion that the mindfulness traits produce changes in forgiveness without the need for a decrease in rumination. However, this decrease in rumination is a prerequisite for forgiveness towards other people.

Our results confirm the relationship between the effects of mindfulness in reducing anger rumination and forgiveness towards oneself and others. This is in line with Peters et al. [69] who conclude that mindfulness training reduces anger and aggression through reductions in anger rumination.

Furthermore, in this research it was found that mindfulness thinking has a mediating effect that reduces rumination. In this respect, Borders et al. [68] examined whether rumination could be a mechanism through which mindfulness is associated with less aggression, anger and hostility. Their results suggest that rumination partially mediated the relationship between mindfulness and hostility, and anger. It appears that people with higher levels of mindfulness are less hostile partly because they ruminate less. According to Borders et al. [68], attention to the present moment contrasts with the uncontrollable circular thoughts experienced when ruminating. This intentional attention to the ‘here and now’ requires cognitive flexibility, as there is much to be aware of at any given time, and in contrast, rumination has been associated with less flexibility. This deficit in flexibility would make it difficult for people with a ruminative style to get out of this mode of thinking [77]. More specifically, Takebe, Takahashi, and Sato [72] found that mindfulness reduced anger rumination and that mindfulness indirectly reduced the anger trait through reduced rumination. Some authors point out that the mindfulness trait predicts less of a rumination trait and anger trait [68,69,72]. 

Coffey and Hartman point out that mindfulness could be beneficial through emotional regulation, detachment and reduced rumination [78]. It can be conceived as a form of attention regulation, where individuals with higher levels of mindfulness are able to consciously redirect their attention away from negative memories and future concerns, and focus on what is happening in the present moment [74]. 

Mindfulness reduces rumination, this being one of the mechanisms by which well-being is increased. The intentional focus on the ‘here and now’ contrasts with the uncontrollable mental fixation on past events that people experience when they ruminate [68]. Mindfulness also encourages a decentralized perspective on inner experiences, allowing individuals to entertain thoughts and emotions without assuming they are facts [74].

## 5. Conclusions

The objectives of this study focused on analyzing the mediating mechanisms by which mindfulness practice in leisure and non-therapeutic contexts reduces anger rumination and increases dispositional forgiveness in a sample of university students. This analysis has allowed advances in our understanding of the underlying mechanisms, which make this practice successful and valuable for our health, and our psychological, emotional and social well-being.

Regarding the possible practical implications of this study, the results obtained confirm a clear relationship between forgiveness and rumination, and how mindfulness practice can improve and reduce the effects of anger rumination. In this sense, introducing this type of training can help to improve our mental and social health. In the educational context, especially in the university context, these constructs are especially important because they increase prosocial behaviors, positive emotions, enrich bonds and promote healthy behaviors.

With regard to limitations, although this study provides evidence that mindfulness practice can contribute to reducing anger and hostility, the context analyzed only showed this for a sample of students in non-therapeutic contexts and taking into account the anger rumination. On the other hand, the results collected here were obtained on the basis of self-report questionnaires; although the scales used have high reliability and proven validity, some studies propose to complement the results using behavioral measures [52] and/or objective monitoring of mindfulness practice [79]. Another limitation is the small sample size and the fact that we did not use probability sampling to test our hypotheses. Therefore, the results of the present study should be interpreted taking these aspects into account.

It should be noted that, while this study provides evidence that mindfulness can contribute to reducing anger and hostility, the context analyzed has only shown this for samples of students in non-therapeutic contexts, and taking into account the anger rumination. However, due to the importance of the results obtained, it is necessary to extend it to samples from other countries, as well as to ruminative states of depression or anxiety.

Based on the results of our study, future research will focus on possible cultural differences in the relationship between anger rumination, forgiveness and mindfulness practice, as well as the study of other mediating variables related to empathy, using behavioral measures. Likewise, these results should be explored in future research and extended to other samples.

## Figures and Tables

**Table 1:** Items, means, standard deviations, skewness, kurtosis, and reliabilities of subscales.

	Items	M	SD	S	K	Cronbach’s Alpha
Five Facet Mindfulness Questionnaire (FFMQ)	1 to 39	3.13	0.48	0.33	0.12	0.805
Observing	1, 6, 11, 15, 20, 26, 31, and 36	3.11	0.79	−0.13	−0.16	0.848
Describing	2, 7, 12, 16, 22, 27, 32, and 37	3.34	0.84	−0.12	−0.43	0.905
Acting with Awareness	5, 8, 13, 18, 23, 28, 34, and 38	3.05	0.70	0.00	−0.05	0.856
Non-judging of Inner Experience	3, 10, 14, 17, 25, 30, 35, and 39	3.16	0.88	−0.20	−0.78	0.805
Non-reactivity to Inner Experience	4, 9, 19, 21, 24, 29, and 33	3.11	0.79	−0.13	−0.16	0.756
Anger Rumination Scale (ARS)	1 to 19	2.35	0.53	−0.14	−0.47	0.925
Angry or Rage Memories	1, 2, 3, 5, and 15	2.50	0.58	−0.25	−0.27	0.821
Understanding the Causes of the Anger	10, 11, 12, and 17	2.58	0.60	−0.39	−0.19	0.713
Thoughts After the Anger	7, 8, 9, 14, 18, and 19	2.35	0.65	−0.14	−0.57	0.851
Thoughts of Revenge	4, 6, 13, and 16	1.91	0.61	0.70	−0.05	0.774
Heartland Forgiveness Scale (HFS)	1 to 18	4.65	0.83	0.22	−0.48	0.872
Self-Forgiveness	1, 2, 3, 4, 5, and 6	4.56	0.95	−0.17	−0.11	0.716
Forgiveness Towards Others	7, 8, 9, 10, 11, and 12	4.72	1.04	−0.23	−0.33	0.821
Situation-Forgiveness	13, 14, 15, 16, 17, and 18	4.66	1.08	0.12	−0.41	0.816

Note. M = Mean; S = Skewness; K = Kurtosis.

## Data Availability

The data and protocols presented in the study are available from https://doi.org/10.17605/OSF.IO/N6RBD.

## Appendix A

**Table A1:** Descriptive statistics of the Heartland Forgiveness Scale (HFS) items.

Item	Mean	SD	Lowest	Highest	Score (% of Sample)						
					1	2	3	4	5	6	7
HFS 1	4.78	1.38	1	7	0.4	8.3	13.6	6.8	39.4	24.2	7.2
HFS 2	3.84	1.64	1	7	6.8	14.8	28.4	11.4	19.7	13.6	5.3
HFS 3	5.38	1.17	1	7	0.4	0.8	7.2	9.1	35.6	29.2	17.8
HFS 4	4.25	1.64	1	7	4.5	10.6	22.0	16.3	21.2	15.9	9.5
HFS 5	4.97	1.14	1	7	0.4	1.9	9.1	16.3	40.9	24.2	7.2
HFS 6	4.31	1.76	1	7	5.7	12.5	20.1	9.1	23.1	18.2	11.4
HFS 7	4.85	1.60	1	7	1.9	7.6	14.4	12.9	22.0	25.8	15.5
HFS 8	5.15	1.24	1	7	1.9	1.1	8.3	9.1	38.6	30.3	10.6
HFS 9	4.45	1.59	1	7	1.5	10.6	22.3	13.6	19.7	23.5	8.7
HFS 10	4.58	1.41	1	7	2.7	6.1	15.5	14.0	34.5	22.3	4.9
HFS 11	4.08	1.55	1	7	3.8	11	28.8	12.9	21.6	17.0	4.9
HFS 12	5.19	1.19	1	7	0.4	3.4	6.8	8.3	38.3	33.0	9.8
HFS 13	4.20	1.76	1	7	6.4	12.9	20.1	14.4	17.0	19.7	9.5
HFS 14	5.03	1.33	1	7	0.4	4.2	11.7	11.0	33.0	28.0	11.7
HFS 15	4.21	1.64	1	7	3.4	12.5	23.9	15.2	18.6	18.2	8.3
HFS 16	5.08	1.26	1	7	0.4	2.3	8.7	17.0	34.5	23.1	14.0
HFS 17	4.42	1.60	1	7	3.8	7.2	20.1	21.2	18.2	18.6	11.0
HFS 18	5.04	1.36	1	7	1.1	4.2	8.7	14.4	32.2	25.8	13.6

**Table A2:** Descriptive statistics of the Anger Rumination Scale (ARS) items.

Item	Mean	SD	Lowest	Highest	Score (% of Sample)			
					1	2	3	4
ARS 1	2.36	0.71	1	4	9.1	50.0	36.7	4.2
ARS 2	2.58	0.68	1	4	3.8	41.3	48.1	6.8
ARS 3	2.56	0.77	1	4	8.0	37.5	45.1	9.5
ARS 4	1.83	0.84	1	4	41.7	37.5	17.0	3.8
ARS 5	2.47	0.85	1	4	13.6	35.2	41.7	9.5
ARS 6	2.48	0.78	1	4	8.3	44.7	37.9	9.1
ARS 7	2.47	0.91	1	4	17.4	29.5	41.7	11.4
ARS 8	2.08	0.90	1	4	31.1	35.6	27.7	5.7
ARS 9	2.66	0.76	1	4	8.0	27.7	54.5	9.8
ARS 10	2.42	0.83	1	4	14.4	37.1	40.5	8.0
ARS 11	2.87	0.81	1	4	5.3	23.9	49.6	21.2
ARS 12	2.76	0.84	1	4	9.5	21.2	53.0	16.3
ARS 13	1.50	0.73	1	4	61.4	29.2	7.2	2.3
ARS 14	2.28	0.90	1	4	24.2	29.5	40.2	6.1
ARS 15	2.54	0.81	1	4	9.8	36.7	42.8	10.6
ARS 16	1.84	0.81	1	4	38.3	43.2	14.8	3.8
ARS 17	2.28	0.81	1	4	17.8	41.3	36.4	4.5
ARS 18	2.14	0.86	1	4	25.0	41.3	28.0	5.7
ARS 19	2.48	0.84	1	4	13.3	34.8	42.8	9.1

**Table A3:** Descriptive statistics of the Five Facet Mindfulness Questionnaire (FFMQ) items.

Item	Mean	SD	Lowest	Highest	Score (% of Sample)				
					1	2	3	4	5
FFMQ 1	2.52	1.07	1	5	20.5	26.1	38.6	10.2	4.5
FFMQ 2	3.36	1.13	1	5	6.1	17.8	25.4	35.2	15.5
FFMQ 3	3.07	1.19	1	5	9.8	25.4	25.0	27.7	12.1
FFMQ 4	3.15	0.95	1	5	4.9	18.9	37.9	33.0	5.3
FFMQ 5	3.49	1.01	1	5	2.7	11.7	37.9	29.2	18.6
FFMQ 6	2.73	1.16	1	5	16.3	28.0	28.0	21.2	6.4
FFMQ 7	3.43	1.04	1	5	3	15.9	32.6	32.2	16.3
FFMQ 8	2.95	1.00	1	5	6.8	25.8	38.6	22.7	6.1
FFMQ 9	2.89	0.99	1	5	5.7	32.6	33.7	23.1	4.9
FFMQ 10	3.13	1.07	1	5	7.6	20.1	31.8	32.6	8.0
FFMQ 11	2.81	1.29	1	5	20.1	22.3	25.4	20.8	11.4
FFMQ 12	2.57	1.13	1	5	18.9	32.2	26.9	17.0	4.9
FFMQ 13	3.29	1.04	1	5	4.5	16.7	37.9	27.3	13.6
FFMQ 14	2.57	1.23	1	5	23.9	27.3	24.2	17	7.6
FFMQ 15	3.25	1.18	1	5	11.0	13.3	29.5	32.6	13.6
FFMQ 16	2.61	1.08	1	5	15.2	34.1	29.9	15.9	4.9
FFMQ 17	3.07	1.08	1	5	7.6	23.5	31.8	28.8	8.3
FFMQ 18	2.70	1.07	1	5	13.3	33.7	26.5	23.1	3.4
FFMQ 19	2.91	1.07	1	5	10.2	24.2	36.4	22.3	6.8
FFMQ 20	3.24	1.15	1	5	9.8	14.0	31.4	31.4	13.3
FFMQ 21	3.21	1.00	1	5	3.8	21.2	34.5	31.4	9.1
FFMQ 22	2.50	0.92	1	5	11.7	43.2	30.3	13.3	1.5
FFMQ 23	2.62	1.00	1	5	14.0	31.1	36.7	15.5	2.7
FFMQ 24	2.80	1.04	1	5	11.0	29.2	32.2	23.9	3.8
FFMQ 25	2.81	1.08	1	5	13.3	26.1	31.4	25.0	4.2
FFMQ 26	3.60	1.01	1	5	3.4	10.6	26.1	42.4	17.4
FFMQ 27	3.30	1.08	1	5	6.4	16.7	29.9	34.8	12.1
FFMQ 28	2.73	0.92	1	5	6.8	35.6	39.0	15.2	3.4
FFMQ 29	2.95	1.01	1	5	7.2	26.1	36.7	24.2	5.7
FFMQ 30	2.60	1.20	1	5	20.8	29.9	25	17	7.2
FFMQ 31	3.16	1.22	1	5	10.2	21.2	26.9	26.1	15.5
FFMQ 32	3.17	1.14	1	5	9.1	18.9	29.2	31.4	11.4
FFMQ 33	2.97	1.04	1	5	8.3	24.2	34.8	26.9	5.7
FFMQ 34	2.84	0.89	1	5	6.4	26.1	47	17.4	3.0
FFMQ 35	2.64	1.13	1	5	17.8	29.5	28	19.7	4.9
FFMQ 36	3.54	1.04	1	5	4.9	10.6	25.8	42.8	15.9
FFMQ 37	3.12	1.13	1	5	8.0	23.5	28.8	28.4	11.4
FFMQ 38	2.95	0.97	1	5	7.6	21.2	44.3	22.0	4.9
FFMQ 39	2.83	1.13	1	5	12.1	30.3	26.5	24.6	6.4
